# Beyond doctors and hospitals: Nepal's Female Community Health Volunteer model—A new paradigm for global health

**DOI:** 10.3389/fpubh.2025.1587360

**Published:** 2025-05-09

**Authors:** Animesh Ghimire

**Affiliations:** ^1^Faculty of Medicine, Nursing and Health Science, Monash University, Melbourne, VIC, Australia; ^2^Sustainable Prosperity Initiative Nepal, Kathmandu, Nepal

**Keywords:** Female Community Health Volunteers (FCHVs), Self-Help Groups (SHGs), Nepal, distributed leadership practice, networked approach

## Introduction:embracing a distributed leadership model

Public health leadership is often envisioned as a top-down structure, with experts and officials directing initiatives from centralized positions. However, in many low- and middle-income countries (LMICs), the most effective leadership emerges within the communities. This opinion piece contends that Nepal's Female Community Health Volunteers (FCHVs)—local women chosen by their communities to deliver vital health services and education at the grassroots level (1)—alongside Self-Help Groups (SHGs)—informal associations, primarily composed of women, that promote collective action, savings, and financial activities (2)—represent a transformative approach in public health leadership. This distributed leadership model is essential for achieving health equity in resource-constrained environments (3). This model, characterized by community ownership, local knowledge, and proactive problem-solving, offers a powerful alternative to traditional approaches and demands greater recognition and investment.

## The FCHV: a cornerstone of community-based healthcare

For over three decades, FCHVs have formed the bedrock of Nepal's community health system, which started in 1988 (4). These women, who are selected by and are accountable to their respective communities, transcend the role of mere volunteers; they serve as integral architects of community-embedded health systems. Their involvement reflects a deep engagement with local needs and priorities, enabling them to design and implement health strategies that are tailored to the specific contexts of their communities (5, 6). Formal titles alone do not inherently confer leadership qualities; rather, effective leadership is demonstrated through actions such as connecting families to essential services, mobilizing resources, and adapting national health guidelines to fit local contexts (7). The FCHV program, initially focused on family planning, has expanded organically to encompass maternal and child health, non-communicable disease screening, and even disaster response, demonstrating the adaptability and responsiveness inherent in this distributed leadership approach (4, 8). The expansion of tasks among FCHVs represents more than a mere augmentation of their responsibilities; it clearly demonstrates their proactive involvement in identifying and addressing the dynamic health needs of their communities (9). This initiative highlights the fundamental principles of effective leadership within the realm of public health, emphasizing the critical role that community members play in fostering health equity and promoting wellbeing. They serve as trusted advisors, health educators, and advocates, navigating complex social dynamics and cultural sensitivities to ensure that even the most vulnerable populations receive care (10).

## Synergy in action: FCHVs and self-help groups

**Figure 1:**
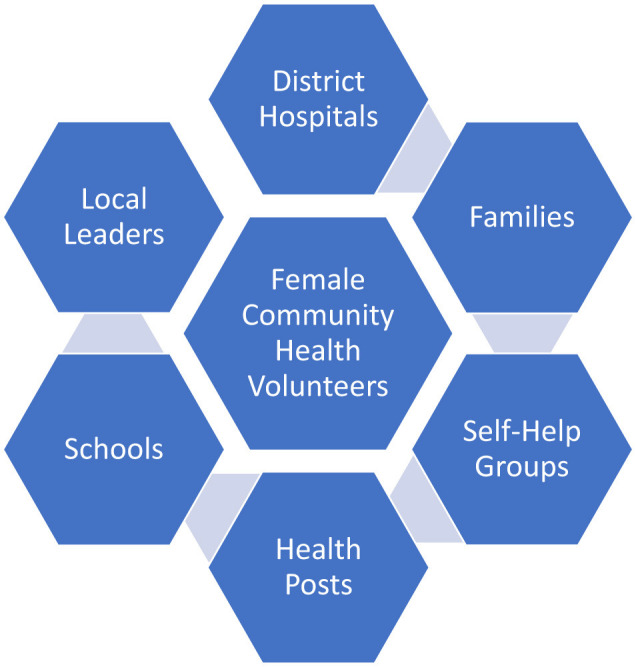
The FCHV as a hub of distributed public health leadership: a networked approach. This diagram illustrates the central role of the Female Community Health Volunteer (FCHV) within a network of community stakeholders. The FCHV acts as a connector and facilitator, linking families, schools, local leaders, health posts, district hospitals, and self-help groups to improve health outcomes. This model demonstrates the power of distributed leadership in a resource-constrained setting.

The effectiveness of the FCHV model is significantly amplified by its interaction with Self-Help Groups (SHGs). This creates a powerful synergy: FCHVs leverage SHG meetings for health education, while SHGs provide a supportive network and mobilize resources for FCHV-led health initiatives (11, 12). FCHVs will help identify and bring the most vulnerable group to the SHGs so that they can also benefit from it (13–15). For example, an FCHV might educate SHG members about the importance of antenatal care, and the SHG, in turn, will provide financial assistance to a pregnant woman who needs to travel to a health facility, often through access to micro-credit programs (16). This interconnectedness—where FCHVs act as trusted sources of health information and SHGs as community mobilizers—exemplifies the distributed leadership model in action. Beyond specific health interventions, this partnership fosters a broader culture of health awareness and empowers women to take control of their own health and wellbeing. The SHGs provide a platform for collective problem-solving, allowing communities to address the social determinants of health, such as poverty and lack of access to education, which often underlie poor health outcomes (16, 17). This vital synergy operates within a broader network where the FCHV serves as a crucial link between families, SHGs, health services, and other community entities, as depicted in Figure 1.

## Navigating challenges: resilience and systemic barriers

The distributed leadership exhibited by FCHVs is not without its challenges. During the 2015 Nepal earthquakes, FCHVs demonstrated remarkable resilience, acting as first responders despite facing personal loss and devastation (5). However, systemic barriers persistently undermine their effectiveness. Inadequate and inconsistent compensation forces many FCHVs to prioritize income-generating activities over their volunteer work, directly impacting the continuity of care (9, 18). Imagine a FCHV, already balancing household responsibilities and childcare, having to trek for hours to reach a remote household, only to receive a meager payment that doesn't even cover her travel expenses (19). This is not a sustainable model, and it devalues the critical role these women play. Furthermore, as the scope of FCHV responsibilities continually expands across various health domains, they face significant overburdening, increasing the risk of burnout and potential dropout (20, 21). The impact of such dropout can be immense; for example, a study on community health workers (CHWs) in Bangladesh calculated significant costs associated with replacing a dropout CHW, compounded by the substantial loss of forgone health services (including health education, maternal care visits, and referrals) for the community during their absence (20).

Several limitations often compound this heavy workload, hampering their ability to reach their full potential, with literacy being a particularly crucial one. Literacy is a fundamental skill that can significantly impact the performance of FCHVs (13, 22). For instance, a lower literacy rate among FCHVs hinders their ability to accurately record health data, understand and implement new health protocols, or effectively communicate complex health information to community members. This can lead to errors in reporting, missed opportunities for health interventions, and reduced effectiveness in health education efforts (9). The limited ongoing training, particularly in rapidly evolving fields such as non-communicable disease management and disaster preparedness, constrain FCHVs' capacity to comprehensively address complex health needs (23). Crucially, inadequate or inconsistent supervision, often lacking a supportive and mentoring approach, further hinders FCHV effectiveness and program success (24), as highlighted by the previous study where women were hesitant to contact FCHVs due to concerns over their competency and literacy skills (25). Significant external and environmental barriers further exacerbate these internal limitations related to skills, support, and community trust. For example, the lack of adequate transportation and communication infrastructure in remote areas isolates these vital community leaders, hindering their ability to connect with health facilities and access support (26). These challenges are not merely logistical hurdles; they are fundamental barriers to the leadership potential of FCHVs and the effectiveness of the distributed leadership model.

## Discussion: investing in a paradigm shift

The FCHV model, particularly in its synergistic relationship with SHGs, offers a compelling example of distributed public health leadership. It demonstrates that effective leadership can, and often must, emerge within communities, driven by local knowledge, trust, and a deep understanding of the context (6). This model is not simply a pragmatic solution for resource-constrained settings; it represents a fundamental shift in how we conceptualize and foster public health leadership. It challenges the traditional, top-down approach that often overlooks the expertise and agency of community members.

Several key implications arise from this analysis. First, the FCHV model should be recognized not as a stop-gap measure, but as a core component of a resilient and equitable health system. This requires a move away from viewing FCHVs as simply “volunteers” and toward recognizing them as essential health workers deserving of fair compensation, comprehensive training, structured supportive supervision, and ongoing support (27, 28). Supportive supervision, focusing on collaborative problem-solving, mentorship, and two-way communication rather than purely administrative oversight, is critical for maintaining motivation and quality (29, 30). The establishment of a formal “FCHV Leadership Academy,” offering advanced training in leadership, advocacy, community mobilization, and the use of digital health tools, could significantly enhance their capacity and impact. Such an academy could also serve as a platform for FCHVs to share their experiences, learn from each other, and develop innovative solutions to local health challenges.

Second, the synergistic relationship between FCHVs and SHGs highlights the importance of fostering community-based networks and supporting collective action. Investing in the development and strengthening of SHGs can amplify the impact of FCHVs and contribute to broader social and economic development (31). This includes providing SHGs with access to resources, training, and opportunities to participate in decision-making processes related to health and development.

Third, the challenges faced by FCHVs underscore the need for systemic change. Addressing issues of compensation, training, supportive supervision, and infrastructure is not merely about improving the FCHV program; it is about creating an enabling environment for distributed leadership to thrive. This also necessitates exploring innovative models to mitigate the risk of overburdening FCHVs. Strategies such as clearer role definition, task simplification, potentially adopting a household-based assignment model where FCHVs provide routine health oversight and support to entire families, rather than solely tracking individual patients with specific conditions (32), better integration with facility-based services to reduce duplication, and appropriate use of digital tools for specific tasks (e.g., data reporting, receiving alerts) should be investigated and implemented to ensure the role remains sustainable and effective (33, 34). This requires commitment from policymakers, health institutions, and international organizations to invest in community-based solutions and empower local actors. This includes advocating for policies that support fair wages for community health workers, investing in infrastructure development in rural areas, and ensuring that FCHVs have access to the resources and support they need to perform their roles effectively (35). Furthermore, it is crucial to address the gender inequalities that often limit women's participation in leadership roles. One promising avenue for empowering FCHVs and overcoming some of these limitations is the application of digital technologies and improved data management. Leveraging digital tools can enhance FCHVs' efficiency, improve data collection and reporting, and facilitate better communication within the health system. Providing the FCHV with a preloaded smartphone, for instance, can improve data quality, allow for real-time monitoring, and improve communication among FCHVs (36).

The Nepali Female Community Health Volunteer model represents a transformative approach to public health leadership with global implications. It provides “lessons” and a proven blueprint for building resilient, community-driven health systems that prioritize equity and responsiveness. Policymakers, international health organizations, and governments in LMICs must move beyond rhetoric and actively invest in this distributed leadership model. This means providing fair compensation and comprehensive training for community health workers, fostering synergistic partnerships with community-based organizations like Self-Help Groups, and leveraging digital technologies to empower these vital frontline actors. Embracing this model is not simply about improving health outcomes but fundamentally reshaping health systems to be more equitable, just, and, ultimately, more effective. It is about recognizing and supporting the true architects of community health: the individuals who live and work within them.
